# Association of prenatal exposure to benzodiazepines and child internalizing problems: A sibling-controlled cohort study

**DOI:** 10.1371/journal.pone.0181042

**Published:** 2017-07-26

**Authors:** Ragnhild E. Brandlistuen, Eivind Ystrom, Sonia Hernandez-Diaz, Svetlana Skurtveit, Randi Selmer, Marte Handal, Hedvig Nordeng

**Affiliations:** 1 Pharmacoepidemiology and Drug Safety Research Group, School of Pharmacy, PharmaTox Strategic Research Initiative, Faculty of Mathematics and Natural Sciences, University of Oslo, Oslo, Norway; 2 Norwegian Institute of Public Health, Oslo, Norway; 3 Department of Psychology, Faculty of Social Sciences, University of Oslo, Oslo, Norway; 4 Department of Epidemiology, Harvard T.H. Chan School of Public Health, Boston, Massachusetts, United States of America; Shinshu University School of Medicine, JAPAN

## Abstract

**Background:**

During pregnancy, many women experience sleep problems and anxiety that require treatment. The long-term safety for the child of maternal benzodiazepine (BZD) and z-hypnotic use during pregnancy remains controversial.

**Method:**

We conducted a cohort and a sibling control study using data from the Norwegian Mother and Child Cohort Study. Data on use of BZD and z-hypnotics, internalizing and externalizing outcomes, and covariates were collected from mothers at gestational weeks 17 and 30 and when children were 0.5, 1.5, and 3 years of age. The total sample consisted of 71,996 children (19,297 siblings) at 1.5 years and 55,081 children (13,779 siblings) at 3 years. Short-term use was defined as use in one pregnancy period only. Long-term use was defined as use in two or more pregnancy periods. Linear full cohort random-effect and sibling-matched fixed-effect regression models were used to compare internalizing and externalizing behavior in children prenatally exposed compared to those unexposed in the full cohort of pregnancies accounting for family clusters, as well as within sibling clusters comparing pregnancies with discordant exposures. Propensity score (PS) adjustment included variables on indication for use (sleep problems, symptoms of anxiety and depression) and other potential confounding factors.

**Results:**

Long-term prenatal exposure to BZD or z-hypnotics was associated with increased internalizing behavior in crude cohort analyses and at age 1.5 years after PS adjustment in sibling-matched fixed-effect models [β 0.60, 95% confidence interval 0.17–0.95]. Analyses on specific drug groups showed that prenatal exposure to BZD-anxiolytics was associated with increased internalizing problems at both 1.5 years [β 0.25, 0.01–0.49] and 3 years [β 0.26, 0.002–0.52] while exposure to z-hypnotics was not associated with any adverse outcomes after adjustment.

**Conclusion:**

The findings suggest a moderate association between BZD-anxiolytic exposure and child internalizing problems that is not likely due to stable familial confounding factors.

## Introduction

Up to 20% of women are estimated to experience sleep problems and symptoms of anxiety during pregnancy [1, 2]. Between 1 and 3% of Norwegian women use benzodiazepine (BZD) or z-hypnotic drugs to treat anxiety disorders or insomnia while pregnant [3]. These medications have sedative, hypnotic, anxiolytic, anticonvulsant, and muscle-relaxing effects depending primarily on how long they act on the gamma-aminobutyric acid (GABA_A_) receptor [4]. BZDs and z-hypnotics cross the placenta and have the potential to bind to receptors in the developing embryonal/fetal central nervous system [5]. It has been proposed that exposure to medications that mimic GABA at GABA_A_ receptors also trigger apoptotic neurodegeneration in the developing brain [6].

In the 1980s, a number of rat studies consistently showed that prenatal exposure to BZDs resulted in behavioral deficits and altered brain function [7–11]. Withdrawal syndrome and floppy infant syndrome have been reported in babies after maternal use of BZDs towards the end of pregnancy [12–15]. Results of previous studies on mental health outcomes in the offspring following prenatal exposure to BZDs or z-hypnotic drugs have been conflicting [16–18]. A sibling-matched study found no association with behavioral deviation in children at age 8–12 months after prenatal exposure to medazepam [16]. In contrast, a study on 1.5-year-old children showed reduced personal-social behavior abilities in children exposed to BZDs [17]. The only published investigation of behavioral outcomes in children older than 1.5 years following prenatal exposure to BZDs was a retrospective study based on 15 exposed children, which found no effects on behavior at the age of 9–10 years [18]. None of these studies controlled for indication for use in the mother.

Well-designed observational studies and randomized controlled trials (RCTs) have been called for to resolve these conflicting findings [19], but because RCTs involving pregnant women are ethically problematic, observational studies addressing confounding by indication and other potential confounders take precedent [20]. In this study, using data from the Norwegian Mother and Child cohort study (MoBa), we aimed to evaluate whether prenatal exposure to BZDs and z-hypnotics affects internalizing or externalizing behavior problems in children at ages 1.5 and 3 years. Such problems are characterized respectively by negative mood states and behavioral inhibition and by behavioral disinhibition [21]. Genetic or familial environmental determinants might result in an increased risk of both mood disorders and anxiety in the mother and behavioral problems in the infant, and living with a mother with these disorders could affect a child’s behavior. To reduce confounding by family conditions, we performed sibling-matched analyses using fixed-effect (FE) models as a conservative approach to adjust for shared familial confounding. To control for indications that vary over time and could confound the sibling comparisons, we used propensity scores (PS) to adjust for indication for use (sleep problems, symptoms of anxiety and depression) and other potential confounding factors.

## Materials and methods

### Data collection

Data were extracted from the ongoing MoBa study conducted by the Norwegian Institute of Public Health. MoBa is a prospective population-based cohort study following over 100,000 pregnancies [22]. Enrollment in the study started in 1999 and was completed in 2008. The participation rate at first assessment was 40.6%. Mothers reported a range of sociodemographic, medical, and psychological information by filling out questionnaires at gestational weeks (gw) 17 and 30 and at 6, 18, and 36 months postpartum (with response rates of 95%, 92%, 87%, 77%, and 62%, respectively). We used the seventh version of the dataset, which is quality-assured and was released in 2013 [http://www.fhi.no/moba-en].

The Medical Birth Registry of Norway (MBRN) includes information on pregnancy, delivery, and neonatal health on all births in Norway [23]. The MoBa and MBRN data were linked via personal identification numbers.

The research project is approved by the Regional Committees for Medical and Health Research Ethics (REK) (2015/1897), and the MoBa study has a license from the Norwegian Data Inspectorate. Written informed consent was obtained from all participants.

### Study population

**Fig 1:**
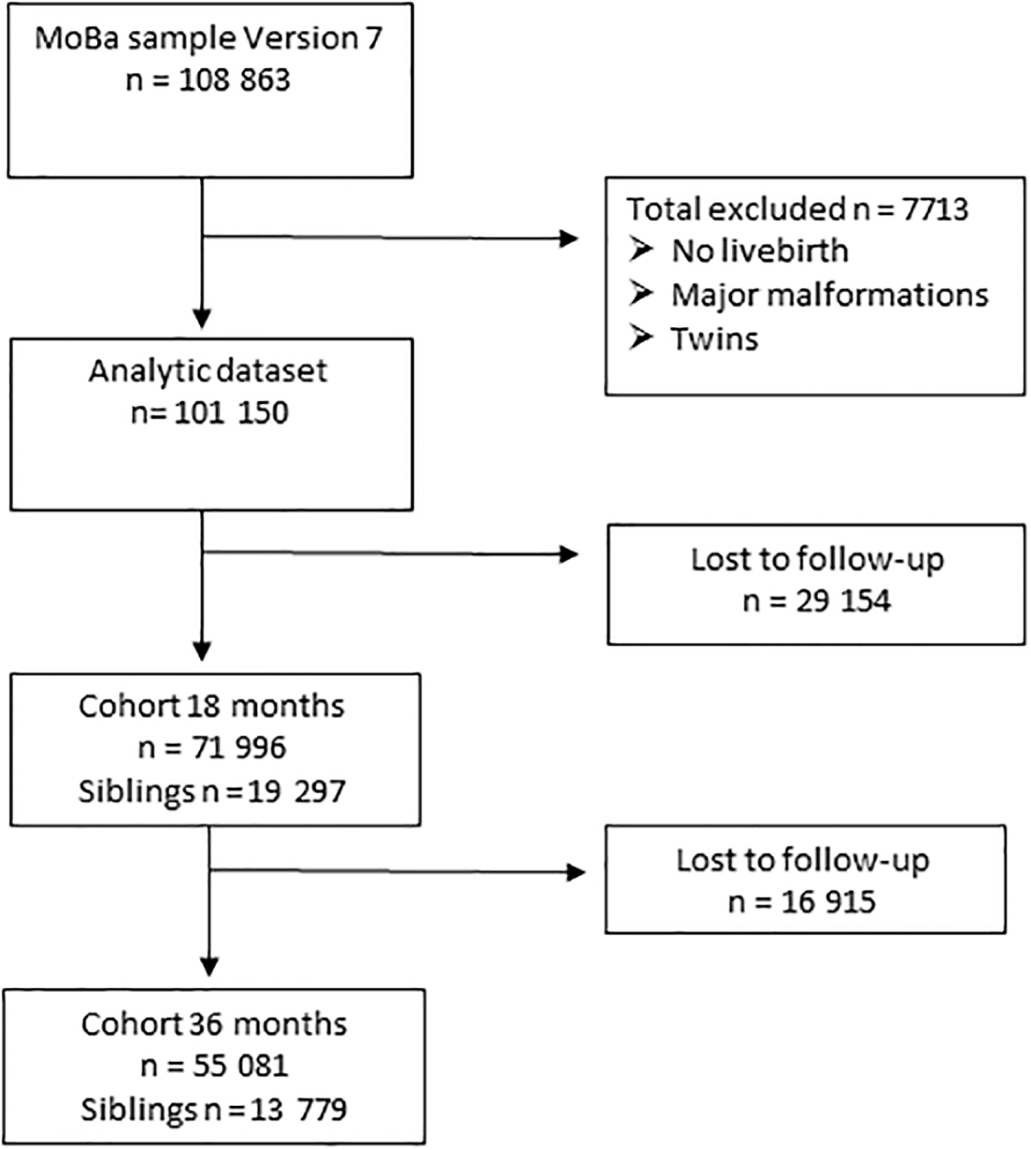
Flow chart of participants.

This study was based on data for 71,996 children whose mothers had returned the 1.5-year follow-up questionnaire and 55,081 children whose mothers had returned the 3-year follow-up questionnaire. Within this cohort, a total of 19,297 siblings were included at 1.5 years and 13,779 siblings at 3 years. All live-born singletons were included in the study population; twins and triplets (N = 4305) and infants born with major malformations (N = 2970) (Fig 1) were excluded.

### BZD and z-hypnotics exposure

BZDs included drugs classified in Anatomical Therapeutic Chemical (ATC) groups N05BA (diazepam, oxazepam, alprazolam), N05CD (nitrazepam, midazolam, flunitrazepam), N03AE01 (clonazepam). Z-hypnotics included drugs classified in the ATC group N05CF (zopiclone and zolpidem). Drug classification was based on the ATC classification system developed by the WHO Collaborating Centre for Drug Statistics Methodology [24]. Women reported information about illnesses they experienced throughout their pregnancy and medication used for these illnesses in two prenatal (17 gw, 30 gw) and one postnatal questionnaire (6 months postpartum) covering three time periods: 0 to 13 gw and 14 to 29 gw, and from 30 gw to birth. The women were defined as users of BZDs and z-hypnotics if they had reported use of any BZD or z-hypnotic drug (ATC groups N05BA, N05CD, N05CF, and N03AE01) during pregnancy on at least one of the three questionnaires. Short-term use was defined as reported use in one pregnancy period only (0–13, 14–29, 30–birth), and long-term use was defined as reported use in two or more pregnancy periods. Separate analyses were also performed for the most commonly reported drug groups: BZD-anxiolytics and z-hypnotics. The drug groups of BZDs and z-hypnotics used, as well as the number of siblings discordantly exposed, are described in Table 1.

**Table 1:** Number of pregnant women reporting use of BZDs or z-hypnotics^a^.

Drug group	Number of children	Number of discordant siblings	Number of children	Number of discordant siblings
	1.5 year	1.5 year	3 years	3 years
	N = 71,996 (%)	Of N = 19,297	N = 55,081 (%)	Of N = 13,779
Any benzodiazepine or z-hypnotics	577 (0.80)	183	440 (0.80)	145
*Duration of use*				
Short-term use^b^	463 (0.64)	157	351 (0.64)	123
Long-term use^c^	114 (0.16)	26	89 (0.16)	22
*Drug subgroups*				
BZD-anxiolytics	315 (0.44)	108	236 (0.43)	84
BZD-hypnotics	44 (0.06)	12	35 (0.06)	8
BZD-anti-epileptics	19 (0.03)	6	12 (0.02)	8
Z-hypnotics	241 (0.34)	75	192 (0.35)	59

Abbreviations: BZD, benzodiazepines; z-hypnotics, benzodiazepine-related hypnotics

^a^ Some women may have reported use related to several drug groups.

^b^ Short-term use was defined as reported use in one pregnancy period only.

^c^ Long-term use was defined as reported use in two or more pregnancy periods.

### Behavior outcomes

The Child Behavior Checklist (CBCL), a validated and widely used measure of child behavior, was used at 1.5 and 3 years [25]. In MoBa, a shortened version of the CBCL was used; it covers both externalizing (i.e., aggression, attention problems) and internalizing behavior (i.e., anxiousness, emotional reactivity, somatic complaints) and is representative of the full scale [26]. Items were selected by a team of four clinical and developmental psychologists, based on clinical and theoretical standards, as well as empirical representativeness (high factor loadings) for behavior problems. The questionnaire consisted of 13 items at 1.5 years and 21 items at 3 years, covering externalizing behavior and internalizing behavior. Externalizing behavior was measured by eight items at 1.5 years and 12 items at 3 years, and internalizing behavior was measured by five items at 1.5 years and nine items at 3 years. These domains predict later psychopathology in children and adolescents [27]. The questions were answered on a 3-point scale, as follows: 1 = not true; 2 = somewhat or sometimes true; and 3 = very true or often true. Mean scores were calculated for externalizing and internalizing behaviors, respectively, and standardized z-scores then were computed for both behavioral outcomes.

### Potential confounders

Confounders were identified by means of a literature review and through use of directed acyclic graphs [28] [29] (http://www.dagitty.net/dags.html) [30]. All potential confounders considered are listed in Tables 2 and 3. Information regarding maternal age and parity was retrieved from the MBRN. Information on maternal years of formal education, marital status, pre-pregnancy BMI, sleep problems, chronic disease, and use of folic acid supplements in early pregnancy was retrieved from the first pregnancy questionnaire. Information on alcohol use, illicit drug consumption during pregnancy, and smoking was retrieved from all three pregnancy questionnaires.

**Table 2:** Maternal characteristics by use of BZDs and z-hypnotics during pregnancy in the 1.5 year MoBa sample.

	Use of BZDs and z-hypnotics			
	1.5-year sample			
	No		Yes	
	N = 71,419		N = 557	
	N	%	N	%
Maternal age in years				
<25	6,948	9.7	41	7.1
25–29	23,807	33.3	149	25.8
30–34	28,130	39.4	237	41.1
≥35	12,534	17.6	150	26.0
Maternal formal education in years				
<12	4,229	6.3	55	10.2
12	17,931	26.6	126	23.3
13–16	28,576	42.4	226	41.9
≥17	16,594	24.7	133	24.6
Marital status				
Married or cohabitant	68,416	98.1	518	93.0
Single	1,357	1.9	36	7.0
Maternal BMI				
<25	47,712	69.2	398	71.5
25–29	14,936	21.7	111	19.9
30–34	4,618	6.7	41	7.4
≥35	1,643	2.4	7	1.3
Parity				
0	32,999	46.2	282	48.9
1	24,914	34.9	171	29.6
≥2	13,506	18.9	124	21.5
Maternal smoking				
No	66,159	92.6	486	84.2
Yes	5,260	7.4	91	15.8
Maternal alcohol use				
No	32,563	45.8	176	30.7
Yes, occasionally	35,958	50.6	340	59.2
Yes, often	2,509	3.6	58	10.1
Maternal symptoms of anxiety/depression 17 gw ^a^				
No	67,105	94.0	456	79.0
Yes	4,314	6.0	121	21.0
Maternal symptoms of anxiety/depression 30 gw ^a^				
No	67,377	94.3	449	77.8
Yes	4,042	5.7	128	22.2
Lifetime history of major depression^b^				
No	56,089	78.5	280	48.5
Yes	15,330	21.5	297	51.5
Maternal sleep problems				
No	60,361	84.5	328	56.8
Yes	11,058	15.5	249	43.2
Maternal use of illicit drugs^c^				
No	71,205	99.7	538	96.6
Yes	214	0.3	19	3.4
Maternal opioid use in pregnancy				
No	70,013	98.0	499	86.5
Yes	1,406	2.0	78	13.5
Maternal SSRI use in pregnancy				
No	70,780	99.1	489	84.8
Yes	639	0.9	88	15.3
Maternal antipsychotic use in pregnancy				
No	70,780	99.1	489	84.8
Yes	639	0.9	88	15.3
Maternal triptan use in pregnancy				
No	70,778	99.1	559	96.9
Yes	641	0.9	18	3.1
Maternal NSAID use in pregnancy				
No	66,542	93.2	485	84.1
Yes	4,877	6.8	92	15.9
Maternal use of paracetamol (acetaminophen)				
No use	39,294	59.3	224	41.3
1 pregnancy period	24,757	37.3	235	43.4
2 or 3 pregnancy periods	2,244	3.2	83	15.3
Maternal folic acid supplements in early pregnancy				
No	22,830	32.0	183	31.7
Yes	48,589	68.0	394	68.3
Maternal chronic disease^d^				
No	64,412	90.2	474	82.2
Yes	7,007	9.8	103	17.8

SSRI, selective serotonin reuptake inhibitors; NSAID, nonsteroidal anti-inflammatory drug

^a^ Mean score of >2 on the Hopkins Symptom Checklist (SCL-5)

^b^ Measured in pregnancy week 17; defined as ever having three co-occurring symptoms of depression for at least 2 weeks and admitting to sad mood

^c^ Illicit drug use included hashish, amphetamine, ecstasy, cocaine, or heroin

^d^ Chronic disease included asthma, diabetes treated with insulin, Crohn’s disease, arthritis, lupus, epilepsy, multiple sclerosis, or cancer

**Table 3:** Maternal characteristics by use of BZDs and z-hypnotics during pregnancy in the 3-year MoBa sample.

	Use of BZDs and z-hypnotics			
	3-year sample			
	No		Yes	
	N = 54,641		N = 440	
	N	%	N	%
Maternal age in years				
<25	5,058	9.2	31	7.0
25–29	18,170	33.3	117	26.6
30–34	21,720	39.8	175	39.8
≥35	9,693	17.7	117	26.6
Maternal formal education in years				
<12	2,902	5.6	37	9.0
12	13,112	25.4	89	21.5
13–16	22,467	43.5	178	43.1
≥17	13,164	25.5	109	26.4
Marital status				
Married or cohabitant	52,455	98.2	396	93.2
Single	976	1.8	29	6.8
Maternal BMI				
<25	36,649	69.4	292	68.9
25–29	11,439	21.7	92	21.7
30–34	3,498	6.6	33	7.8
≥35	1,249	2.4	7	1.7
Parity				
0	25,851	47.3	229	52.0
1	18,889	34.6	120	27.3
≥2	9,901	18.1	91	20.7
Maternal smoking				
No	50,852	93.1	376	85.5
Yes	3,789	6.9	64	14.5
Maternal alcohol use				
No	25,671	47.3	147	33.5
Yes, occasionally	26,712	49.2	251	57.2
Yes, often	1,946	3.6	41	9.3
Maternal symptoms of anxiety/depression 17 gw ^a^				
No	51,510	94.3	344	78.2
Yes	3,131	5.7	96	21.8
Maternal symptoms of anxiety/depression 30 gw ^a^				
No	51,662	94.6	340	77.3
Yes	2,979	5.5	100	22.7
Lifetime history of major depression^b^				
No	43,096	78.9	213	48.4
Yes	11,545	21.1	227	51.6
Maternal sleep problems				
No	46,014	84.2	246	55.9
Yes	8,627	15.8	194	44.1
Maternal use of illicit drugs^c^				
No	51,071	99.8	395	96.8
Yes	114	0.2	13	3.2
Maternal opioid use in pregnancy				
No	53,558	98.0	392	89.1
Yes	1,083	2.0	48	10.9
Maternal SSRI use in pregnancy				
No	54,163	99.1	363	82.5
Yes	478	0.9	77	17.5
Maternal antipsychotic use in pregnancy				
No	54,546	99.8	416	94.6
Yes	95	0.2	24	5.4
Maternal triptan use in pregnancy				
No	54,120	99.0	426	96.8
Yes	521	1.0	14	3.2
Maternal NSAID use in pregnancy				
No	50,871	93.1	367	83.4
Yes	3,770	6.9	73	16.6
Maternal use of paracetamol (acetaminophen)				
No use	27,971	54.0	151	35.2
1 pregnancy period	13,933	26.9	124	28.9
2 or 3 pregnancy periods	9,884	19,1	154	25.9
Maternal folic acid supplements in early pregnancy				
No	16,250	29.7	124	28.2
Yes	38.391	70.3	316	71.8
Maternal chronic disease^d^				
No	49,282	90.2	361	82.1
Yes	5,359	9.8	79	17.9

SSRI, selective serotonin reuptake inhibitors; NSAID, nonsteroidal anti-inflammatory drug

^a^ Mean score of >2 on the Hopkins Symptom Checklist (SCL-5)

^b^ Measured in pregnancy week 17; defined as ever having three co-occurring symptoms of depression for at least 2 weeks and admitting to sad mood

^c^ Illicit drug use included hashish, amphetamine, ecstasy, cocaine, or heroin

^d^ Chronic disease included asthma, diabetes treated with insulin, Crohn’s disease, arthritis, lupus, epilepsy, multiple sclerosis, or cancer

Maternal symptoms of depression and anxiety during the pregnancy were reported via a validated short version of the Hopkins Symptom Checklist, the SCL-5, at 17 and 30 gw [31]. The 5-item version (SCL-5) has a 0.92 correlation with the original 25-item version. Items in the SCL are scored on a Likert scale ranging from 1 (not at all bothered) to 4 (very much bothered), and scores were calculated as the mean value of the five items. The presence of symptoms of anxiety and depression during pregnancy was defined as 85^th^ percentile or above. Mothers also reported their lifetime history of major depression by answering the lifetime occurrence of five key depressive symptoms chosen from the nine symptomatic criteria for Major Depression in the Diagnostic and Statistical Manual of Mental Disorders III, Revised: sad mood, change in appetite, loss of energy, feelings of guilt or worthlessness, and problems in concentration. They were then asked whether any three of these symptoms co-occurred in their life for at least 2 weeks. As previously recommended, individuals who responded positively to this item and admitted to sad mood were considered to have reported a lifetime history of major depression [32].

Maternal concomitant use of other drugs (opioids, selective serotonin reuptake inhibitors (SSRIs), antipsychotics, triptans, nonsteroidal anti-inflammatory drugs (NSAIDs), and paracetamol) were reported at 17 and 30 gw and 6 months after birth. Because we previously found an association between long term paracetamol exposure and behavior problems in the child we differentiated between short and long term paracetamol use[33].

### Statistical methods

First, we used random-effects linear regression models to examine crude effect estimates on behavior outcomes at 1.5 and 3 years following prenatal exposure to any BZD or z-hypnotics, short term or long term, for the full MoBa cohort. Random-effects models account for family clusters in the sample by producing standard errors that adjust for dependence between siblings. Separate analyses were performed for BZD-anxiolytics and z-hypnotics.

Second, we performed PS-adjusted random-effect linear regression analyses by including PS as covariates in the analyses. PS were determined with logistic regression models within the complete cohort at 1.5 years and 3 years separately, in which use of any BZD or z-hypnotics was the outcome variable and the predictors were the variables previously selected (see Tables 2 and 3) as confounders during multivariable model building [34].

Third, we restricted the sample to multiparous mothers and then carried out sibling-matched FE linear regression using the xtreg function in STATA 14 for FE linear models. As default in STATA, we used the observed information matrix to calculate 95% confidence intervals. In the FE model, each sibling’s score on the outcome variables (externalizing and internalizing behaviors) was subtracted from the average scores of all siblings in his/her family, and each sibling’s value of the predictor (prenatal exposure to BZDs or z-hypnotics) was subtracted from the average values of all siblings in his/her family. The new outcome variables were regressed on the new exposure variables. The sibling FE models thereby controlled for potential bias by unobserved variables that were fixed within families (constant across siblings), such as shared family environments or genetic transmission of risk (e.g., maternal personality and intellectual ability). The effect estimates are within families of siblings, and only siblings discordant on exposure and outcome contributed any information. Finally, we carried out PS-adjusted FE models to adjust for the potential confounding factors varying between siblings. All analyses were done using Stata Version 14 (Stata Corporation, College Station, TX, USA).

## Results

In total, 0.8% of the children were exposed to BZDs and z-hypnotics during pregnancy (N_1.5 years_ = 577 of 71,996; N_3 years_ = 440 of 55,081) (Fig 1).

### Internalizing behavior problems

Table 4 shows the results of full cohort (random-effect) and sibling-matched (FE) linear regression models on internalizing behavior problems predicted by *in utero* exposure to BZDs and z-hypnotics, short term, long term, and for BZD-anxiolytics and z-hypnotics separately. Short-term exposure to BZDs and z-hypnotics was associated with increased internalizing behavior problems in crude analyses at both 1.5 and 3 years of child age. These effects were attenuated when accounting for the factors associated with maternal use of BZDs and z-hypnotics during pregnancy by PS score adjustment. For long-term exposure, increasing associations were observed in the sibling-matched models at 1.5 years and were not attenuated after PS score adjustment. At 3 years, crude effects were observed but decreased and were not significant in the PS-adjusted model. However, in the analyses on exposure to BZD-anxiolytics specifically, effect estimates from the sibling-matched model on internalizing behavior remained elevated after adjustment at both 1.5 and 3 years. For z-hypnotics, crude increased internalizing behavior problems were attenuated after adjusting for PS scores and familial confounding in the sibling-matched models.

**Table 4:** Full cohort and sibling-matched linear regression models: Parameters for differences in internalizing behavior score predicted by *in utero* exposure to BZDs and z-hypnotics.

		Internalizing behavior		
		1.5 years		3 years
	N	β (95% CI)	N	β (95% CI)
*Any BZDs and z-hypnotics*				
Short term^a^				
Crude cohort sample (re)	71,649	0.12 (0.06, 0.19)	54,741	0.18 (0.10, 0.25)
PS-adjusted cohort sample (re)^b^	60,964	0.07 (0.001, 0.14)	47,767	0.08 (-0.02, 0.16)
PS-adjusted sibling sample (re) ^b^	18,193	0.12 (-0.01, 0.25)	13,199	0.15 (-0.01, 0.29)
Sibling-matched sample (fe)	19,397	0.10 (-0.08, 0.29)	13,711	0.12 (-0.07, 0.32)
PS-adjusted sibling-matched sample (fe) ^c^	18,193	0.13 (-0.06, 0.32)	13,199	0.12 (-0.08, 0.32)
Long term^a^				
Crude cohort sample (re)	71,649	0.11 (-0.03, 0.24)	54,741	0.21 (0.06, 0.36)
PS-adjusted cohort sample (re) ^b^	60,964	0.01 (-0.13, 0.15)	47,767	0.04 (-0.12, 0.20)
PS-adjusted sibling sample (re) ^b^	18,193	0.21 (-0.07, 0.49)	13,199	0.06 (-0.25, 0.36)
Sibling-matched sample (fe)	19,397	0.46 (0.08, 0.85)	13,711	0.21 (-0.19, 0.62)
PS-adjusted sibling-matched sample (fe) ^c^	18,193	0.60 (0.17, 0.95)	13,199	0.15 (-0.26, 0.56)
*Stratified analyses on drug groups*^d^				
BZD-anxiolytics				
Crude cohort sample (re)	71,649	0.09 (0.003, 0.17)	54,741	0.24 (0.14, 0.34)
PS-adjusted cohort sample (re) ^b^	60,964	0.05 (-0.04, 0.14)	47,767	0.13 (0.03, 0.24)
PS-adjusted sibling sample (re) ^b^	18,193	0.24 (0.07, 0.41)	13,199	0.23 (0.03, 0.43)
Sibling-matched sample (fe)	19,397	0.26 (0.02, 0.49)	13,711	0.36 (0.17, 0.55)
PS-adjusted sibling-matched sample (fe) ^c^	18,193	0.25 (0.01, 0.49)	13,199	0.26 (0.002,0.52)
Z-hypnotics				
Crude cohort sample (re)	71,649	0.14 (0.05, 0.24)	54,741	0.15 (0.05, 0.25)
PS-adjusted cohort sample (re) ^b^	60,964	0.05 (-0.05, 0.15)	47,767	0.02 (-0.09, 0.13)
PS-adjusted sibling sample (re) ^b^	18,193	0.01 (-0.17, 0.19)	13,199	0.03 (-0.17, 0.23)
Sibling-matched sample (fe)	19,397	-0.05 (-0.32, 0.22)	13,711	0.10 (-0.10, 0.30)
PS-adjusted sibling-matched sample (fe) ^c^	18,193	0.02 (-0.26, 0.30)	13,199	0.05 (-0.24, 0.35)

CI, confidence interval; re, random effects; fe, fixed effects; PS: propensity score. Effect sizes (β) for all outcomes should be interpreted as the increase in terms of standard deviations in the outcome variable (Cohen’s d) in exposed children (reference group: unexposed).

^a^ Three pregnancy periods were defined: gw 0–13, 14–29, 30–birth. Short-term use was defined as reported use in one pregnancy period only. Long-term use was defined as reported use in two or more pregnancy periods.

^b^ Adjusted for the propensity for taking BZDs and z-hypnotics during pregnancy, conditional on maternal age, formal education, marital status, pre-pregnancy BMI, parity, smoking, or alcohol use during pregnancy, presence of symptoms of anxiety/depression at gw 17 and/or gw 30, lifetime history of major depression, sleep problems, use of illicit drugs, chronic disease, use of folic acid supplements, and use of other medications during pregnancy (opioids, SSRIs, antipsychotics, triptans, NSAIDs, paracetamol).

^c^ Adjusted for the propensity for taking BZDs and z-hypnotics during pregnancy, conditional on maternal age, parity, alcohol use during pregnancy, presence of symptoms of anxiety/depression gw 17 and/or gw 30, lifetime history of major depression, sleep problems, use of illicit drugs, use of folic acid supplements, and use of other medications during pregnancy (opioids, SSRIs, antipsychotics, triptans, NSAIDs, paracetamol).

^d^ Including both short- and long-term exposure.

### Externalizing behavior problems

Table 5 shows the results of full cohort (random-effect) and sibling-matched (FE) linear regression models for externalizing behavior outcomes predicted by *in utero* exposure to BZDs and z-hypnotics, short term, long term, and for BZD-anxiolytics and z-hypnotics separately. In crude analyses of short-term exposure to BZDs and z-hypnotics, a small increase in externalizing behavior problems was observed at child age 1.5 years, with increasing effect estimates for long-term exposure. Taking PS scores into account, all effects on externalizing behavior problems were attenuated. No effects were observed for externalizing behavior problems in the sibling-matched models for short-term and long-term exposure, accounting for familial confounding, at child age 1.5 or 3 years. In stratified analyses, crude associations with externalizing behavior were observed at 3 years for BZD-anxiolytics and 1.5 years for z-hypnotics. These associations were attenuated when accounting for PS score and familial confounding in the sibling-matched models. At 3 years of age, a beneficial effect of z-hypnotic exposure on externalizing behavior problems was observed after PS score adjustment.

**Table 5:** Full cohort and sibling-matched linear regression models for externalizing behavior predicted by *in utero* exposure to BZDs and z-hypnotics.

		Externalizing behavior		
		1.5 years		3 years
	N	β (95% CI)	N	β (95% CI)
*Any BZDs and z-hypnotics*				
Short term^a^				
Crude cohort sample (re)	71,872	0.08 (0.003, 0.15)	54,709	0.03 (-0.06, 0.12)
PS-adjusted cohort sample (re) ^b^	61,537	0.04 (-0.04, 0.12)	47,741	-0.03 (-0.13, 0.06)
PS-adjusted sibling sample (re)^b^	18,248	0.09 (-0.06, 0.25)	13,191	0.001 (-0.19, 0.19)
Sibling-matched sample (fe)	19,277	0.02 (-0.19, 0.22)	13,701	-0.16 (-0.40, 0.08)
PS-adjusted sibling-matched sample (fe)^c^	18,248	0.02 (-0.19, 0.23)	13,191	-0.15 (-0.76, 0.25)
Long term^a^				
Crude cohort sample (re)	71,872	0.22 (0.07, 0.37)	54,709	0.19 (-0.05, 0.32)
PS-adjusted cohort sample (re)^b^	61,537	0.12 (-0.05, 0.28)	47,741	0.09 (-0.10, 0.28)
PS-adjusted sibling sample (re)^b^	18,248	0.20 (-0.13, 0.53)	13,191	0.13 (-0.25, 0.51)
Sibling-matched sample (fe)	19,277	0.02 (-0.41, 0.44)	13,701	-0.26 (-0.40, 0.08)
PS-adjusted sibling-matched sample (fe)^c^	18,248	-0.03 (-0.46, 0.40)	13,191	-0.25 (-0.76, 0.25)
*Stratified analyses on drug groups* ^d^				
BZD-anxiolytics				
Crude cohort sample (re)	71,872	0.06 (-0.03, 0.16)	54,709	0.13 (0.01, 0.24)
PS-adjusted cohort sample (re)^b^	61,537	0.06 (-0.05, 0.16)	47,741	0.07 (-0.05, 0.20)
PS-adjusted sibling sample (re)^b^	18,248	0.06 (-0.14, 0.25)	13,191	0.31 (0.06, 0.56)
Sibling-matched sample (fe)	19,277	-0.02 (-0.28, 0.25)	13,701	0.07 (-0.24, 0.39)
PS-adjusted sibling-matched sample (fe)^c^	18,248	-0.04 (-0.31, 0.23)	13,191	0.16 (-0.17, 0.48)
Z-hypnotics				
Crude cohort sample (re)	71,872	0.12 (0.02, 0.23)	54,709	0.01 (-0.11, 0.13)
PS-adjusted cohort sample (re)^b^	61,537	0.04 (-0.07, 0.15)	47,741	-0.08 (-0.22, 0.05)
PS-adjusted sibling sample (re)^b^	18,248	0.08 (-0.13, 0.29)	13,191	-0.22 (-0.47, 0.04)
Sibling-matched sample (fe)	19,277	-0.05 (-0.35, 0.24)	13,701	-0.45 (-0.80, 0.10)
PS-adjusted sibling-matched sample (fe)^c^	18,248	-0.03 (-0.34, 0.28)	13,191	-0.52 (-0.87, -0.15)

CI, confidence interval; re, random effects; fe, fixed effects; PS: propensity score. Effect sizes (β) for all outcomes should be interpreted as the increase in terms of standard deviations in the outcome variable (Cohen’s d) in exposed children (reference group: unexposed).

^a^ Three pregnancy periods were defined: gw 0–13, 14–29, 30–birth. Short-term use was defined as reported use in one pregnancy period only. Long-term use was defined as reported use in two or more pregnancy periods.

^b^ Adjusted for the propensity for taking BZDs and z-hypnotics during pregnancy, conditional on maternal age, formal education, marital status, pre-pregnancy BMI, parity, smoking, or alcohol use during pregnancy, presence of symptoms of anxiety/depression at gw 17 and/or gw 30, lifetime history of major depression, sleep problems, use of illicit drugs, chronic disease, use of folic acid supplements, and use of other medications during pregnancy (opioids, SSRIs, antipsychotics, triptans, NSAIDs, paracetamol).

^c^ Adjusted for the propensity for taking BZDs and z-hypnotics during pregnancy, conditional on maternal age, parity, alcohol use during pregnancy, presence of symptoms of anxiety/depression gw 17 and/or gw 30, lifetime history of major depression, sleep problems, use of illicit drugs, use of folic acid supplements, and use of other medications during pregnancy (opioids, SSRIs, antipsychotics, triptans, NSAIDs, paracetamol).

^d^ Including both short- and long-term exposure.

## Discussion

In this large prospective follow-up study including siblings discordantly exposed to BZDs or z-hypnotic drugs, we found that prenatal long-term exposure to BZDs or z-hypnotics was associated with increased internalizing behavior problems among children aged 1.5 years. Of importance, when studied separately, BZD-anxiolytics exposure was associated with increased internalizing problems at both 1.5 and 3 years of follow-up while z-hypnotic exposure was not associated with any adverse outcomes.

These findings are in line with previous studies reporting delayed mental functioning up to the age of 1.5 years after exposure to BZDs [17, 35, 36]. Viggedal et al. observed reduced “personal-social behavior” abilities at child age 1.5 years but found that externalizing problems such as hyperactivity or attention problems were not significantly different from controls [17]. This result is in line with our finding that internalizing, not externalizing, problems were reported. Moreover, our study showed effects up to 3 years of age for BZD-anxiolytics. The only previous study looking at child behavior outcomes beyond the age of 1.5 years found no effects on behavior reported at the ages of 9–10 years [18]. However, no evaluation of internalizing behavior was reported, and the sample consisted of 15 exposed children, limiting the statistical power to detect effects of exposure [18]. It is therefore unclear whether the lack of behavior problems at school age was due to diminishing symptoms, crude measures failing to capture internalizing problems, or low statistical power to detect effects. More prospective follow-up studies with larger sample sizes and measures of internalizing behavior are required to determine the stability of increased internalizing problems beyond preschool age for specific drug groups.

Of note, small associations between z-hypnotic exposure and internalizing behavior problems in the child were attenuated in adjusted analyses taking the indication for use into account. We found no increase in internalizing or externalizing problems in the sibling-matched FE models at any time point for children exposed to z-hypnotic drugs. In contrast, prenatal exposure to z-hypnotics was associated with less externalizing behavior problems at 3 years of age in the sibling-matched analyses. It is possible that using z-hypnotics to reduce sleep problems in pregnancy is beneficial for the child. To our knowledge, no previous study has examined potential long-term effects for the child of prenatal z-hypnotic exposure on behavioral outcomes, and the potential beneficial association between exposure to z-hypnotics and behavioral effects would need to be confirmed by more studies before any conclusions can be made regarding the long-term safety.

The present study has a number of strengths. It uses data from a population-based sample with the possibility of following prospectively more than 400 children prenatally exposed to BZDs and z-hypnotics and a large control group. The richness of the information available enables the opportunity to adjust for many potential confounding factors, including indication for use, which is often not available in conventional studies. Maternal use of BZDs is related to substance abuse and psychiatric problems in the mother [37]. Of importance, the sibling-matched design has a number of advantages in controlling for family factors that are difficult to measure and control for in conventional cohort designs. The intra-class correlation across pregnancies for the outcomes are 0.35 (internalizing) and 0.41 (externalizing) (both P < 0.001) [38], showing that stable factors across pregnancies are potentially strong confounders. Because socioeconomic or genetic factors are likely to confound an association between maternal psychotropic medication use during pregnancy and adverse behavioral outcomes in the child, limiting this type of confounding is an important strength.

The study also has some limitations. First, the sibling-controlled design does not rule out factors that vary between siblings, correlate with exposure within families, or correlate with the outcome [39, 40]. The inclusion of an estimated PS in the sibling analyses accounting for the observed factors shown to be associated with BZD and z-hypnotics use helped control for these factors in the current study. Although reported use of illicit drugs and substance use were controlled for, we cannot rule out confounding by more complex misuse not reported in the questionnaires. Second, the design is sensitive to measurement unreliability. Measurement error in the exposure might lead to an underestimation of the effects [41]. Moreover, the sample size was not large enough to perform analyses for specific trimesters or on single drugs instead of drug groups in a sibling-controlled design. For the same reason, stratified analyses on child gender were not possible, and gender-specific effects could therefore not be explored. The agreement between self-report and dispensed drugs registered in the Norwegian Prescription Database (NorPD) for drugs used intermittently, such as BZD-anxiolytics and z-hypnotics, is lower than for drugs used chronically [42]. This could be because users of BZDs and hypnotics often already have the medications available from previous dispensing. More women reporting BZDs in MoBa were found with a prescription in the NorPD when a longer pre-pregnancy observation period was included [43]. In a study based on a linkage between the MBRN and the NorPD including all women giving birth in Norway, 1.5% were reported to use BZDs. In MoBa, only 0.8% reported use, indicating inclusion of healthier women in that study, which may limit the generalizability of the current findings. However, even though estimates of frequencies can be biased due to selective participation, exposure–outcome associations have not previously differed between MoBa participants and the general population [44]. Gustavson et al. demonstrated using Monte Carlo simulation studies that association estimates are generally quite robust against selective participation even when the frequency estimates are biased [45, 46].

In conclusion, our results indicate that prenatal exposures to BZDs influence internalizing problems in children. Exposure to BZD-anxiolytic or z-hypnotic medications was not associated with increased externalizing problems. Exposure to BZD-anxiolytics, however, was associated with increased internalizing problems in the child, lasting up to 3 years of age. These findings could not be explained by shared familial confounding, reported indication for use, or other measured confounding factors, but residual unmeasured confounding cannot be ruled out.
